# Raloxifene retards the progression of adjacent segmental intervertebral disc degeneration by inhibiting apoptosis of nucleus pulposus in ovariectomized rats

**DOI:** 10.1186/s13018-021-02504-4

**Published:** 2021-06-09

**Authors:** Qi Sun, Xin-Yu Nan, Fa-Ming Tian, Fang Liu, Shao-Hua Ping, Zhuang Zhou, Liu Zhang

**Affiliations:** 1 Department of Orthopedic Surgery, Hebei Medical University, 361 Zhongshan E Rd, Shijiazhuang, Hebei 050000 People’s Republic of China; 2 Medical Research Center, North China University of Science and Technology, Tangshan, People’s Republic of China; 3 Department of Bone and Soft Tissue Oncology, The Third Hospital of Hebei Medical University, Shijiazhuang, People’s Republic of China; 4 Department of Orthopedic Surgery, Emergency General Hospital, Beijing, People’s Republic of China

**Keywords:** Osteoporosis, Animal, Apoptosis, Intervertebral disk, Fusion

## Abstract

**Background:**

Adjacent segmental intervertebral disk degeneration (ASDD) is a major complication secondary to lumbar fusion. Although ASSD pathogenesis remains unclear, the primary cause of intervertebral disk degeneration (IVDD) development is apoptosis of nucleus pulposus (NP). Raloxifene (RAL) could delay ASDD by inhibiting NP apoptosis.

**Methods:**

An ASDD rat model was established by ovariectomy (OVX) and posterolateral spinal fusion (PLF) on levels 4–5 of the lumbar vertebrae. Rats in the treatment groups were administered 1 mg/kg/d RAL by gavage for 12 weeks, following which, all animals were euthanized. Lumbar fusion, apoptosis, ASDD, and vertebrae micro-architecture were evaluated.

**Results:**

RAL maintained intervertebral disk height (DHI), delayed vertebral osteoporosis, reduced histological score, and inhibited apoptosis. The OVX+PLF+RAL group revealed upregulated expression of aggrecan and B-cell lymphoma-2 (bcl2), as well as significantly downregulated expression of a disintegrin and metalloproteinase with thrombospondin motifs 4 (ADAMTS-4), metalloproteinase-13 (MMP-13), caspase-3, BCL2-associated X (bax), and transferase dUTP nick end labeling (TUNEL) staining. Micro-computed tomography (Micro-CT) analysis revealed higher bone volume fraction (BV/TV), bone mineral density (BMD), and trabecular number (Tb.N), and lower trabecular separation (Tb.Sp) in OVX+PLF+RAL group than in the OVX+PLF group.

**Conclusions:**

RAL can postpone ASDD development in OVX rats through inhibiting extracellular matrix metabolic imbalance, NP cell apoptosis, and vertebral osteoporosis. These findings showed RAL as a potential therapeutic target for ASDD.

## Introduction

Lumbar spinal fusion is an effective surgery to treat spinal diseases as it can remove certain lumbar motions [1]. However, it is associated with caudad and cephalad motions near the fusion site, resulting in a high incidence of adjacent segmental intervertebral disk degeneration (ASDD) [2]. ASDD incurs a huge economic and psychological burden on patients and families, seriously affecting the patient’s quality of life. Nucleus pulposus (NP) constitutes an important component of the intervertebral disk (IVD). NP can form an integrated IVD with cartilage endplate (EP) and annulus fibrosus (AF). Extracellular matrix (ECM) mostly comprises collagens, proteoglycans, and aggrecan that form the gelatinous tissues within the IVD [3]. The degeneration of NP is an indispensable aspect of intervertebral disk degeneration (IVDD) [4].

Increasing evidence shows that estrogen influences the health of IVDD [5, 6]. The estrogen deficiency after menopause has a negative impact on the vertebral body and endplates, which increases the risk of IVDD in the postmenopausal period [6, 7]. The pathogenesis of ASDD is not completely understood. However, the apoptosis of NP, as well as the changes in the nutritional absorption in IVD are speculated to participate in ASDD development. Although inflammation and apoptosis maintain tissue homeostasis in the body, excessive apoptosis of NP cells accelerates the IVDD [8]. According to Che and colleagues [9], suppression of NP cell apoptosis delayed IVD degeneration.

Raloxifene (RAL), a second-generation, non-steroidal, selective estrogen receptor modulator (SERM), modulates bone turnover and the nervous system. In addition, it affects several metabolic processes, such as apoptosis, inflammation, and aging, by functioning as the estrogen agonist or antagonist [10, 11]. Several studies have reported the protective effects of RAL against chondrocyte apoptosis, senescence, and bone loss [12–14]. Although NP is the primary component of IVD, the regulatory role of RAL in NP has remained largely unclear. As apoptosis significantly affects IVDD, we speculated RAL to be implicated in ASDD pathogenesis.

We used an ovariectomy (OVX) and posterolateral spinal fusion (PLF) rat model to study the effects of RAL on ASDD. Besides, this study examined the mechanisms responsible for the RAL function in ASDD.

## Materials and methods

### Experimental design

The present study was approved by the Institutional Animal Care and Use Committee. Altogether, 60, 3-month-old Sprague Dawley (SD) rats (Vital River Experimental Animal Technical Co., Ltd., Beijing) weighing 216 ± 14 g (mean ± SD) were randomized into two groups, including the sham group (sham surgery, *n* = 24) and OVX group (bilateral ovariectomy, *n* = 36). The animals in the sham surgery group were randomly assigned to the sham group (*n* = 12) and PLF group (*n* = 12). The animals in the OVX group were randomly assigned to the OVX group (*n* = 12), OVX+PLF group (*n* = 12), and OVX+PLF+RAL group (*n* = 12). PLF surgery was performed at L4–L5 using a previously described procedure [15, 16]. The rats in the OVX+PLF+RAL group were administered 1 mg/kg/d RAL by gavage for 12 weeks. All rats were euthanized at 12 weeks post-PLF to collect the L3–L6 segment.

The rats were maintained at 21 ± 1 °C and 12-h light/dark cycle conditions and were provided food and water freely (HFK Bioscience Co., Ltd., Beijing).

### Manual palpation and X-ray analysis

The L3–L6 segment fusion was evaluated in the lateral recumbent position by soft radiography (DR7500 System, Kodak, USA). The disk height index (DHI) was determined by this formula: anterior disk height + posterior disk height/anterior vertebral bone height + posterior vertebral bone height. Manual palpation is considered a gold standard for assessing the success of pseudarthrosis formation and fusion [17]. The fusion scores were evaluated according to the criteria established by O’Loughlin et al. [18].

### Micro-CT analysis

For investigating changes in vertebrae, the SkyScan 1176 microcomputed tomography system (80 kV, 313 μA, 18 μm) was used to scan the L5-L6 segments. One inner cylinder with a diameter and length of 1.5 mm and 3 mm, respectively, was chosen to be the region of interest (ROI) in L6 vertebrae at the cephalad level. To evaluate the vertebral trabecular structural parameters, we determined the bone mineral density (BMD), trabecular number (Tb.N), trabecular separation (Tb.Sp), and bone volume fraction (BV/TV).

### Histology and immunohistochemistry examinations

Neutral paraformaldehyde (10%) was used to fix the specimens for 48 h at room temperature. Ethylenediaminetetraacetic acid (EDTA)-2Na (10%) was used for further decalcification for 3 months. Thereafter, each specimen was dehydrated, paraffinized, and embedded. Next, each sample was cut into 8-μm sections for terminal deoxynucleotidyl transferase dUTP nick end labeling (TUNEL) staining, Van Gieson (VG) staining, and immunohistochemical (IHC) analysis. VG and TUNEL staining were performed as per the protocols provided with the BA408A VG kit and MA0224 one-step TUNEL apoptosis kit, respectively. The L5–L6 IVD degeneration was studied using histological scoring [19], which was performed by two independent researchers in a blinded manner.

Expression of caspase 3 (1:100; Gene Tex Inc., USA), ADAMTS-4 (1:100; Abcam Inc, USA), matrix metalloproteinase-13 (MMP-13) (1:500; Boster Co., Ltd., Wuhan, China), aggrecan (1:100; Abcam Inc., USA), BCL2-associated X (bax) (1:200; Abcam Inc., USA), and B cell lymphoma-2 (bcl2) (1:300; Abcam Inc., USA) in NP was detected by IHC. Each section was deparaffinized, rehydrated, and immunostained in succession, followed by 30 min of incubation by pancreatin to retrieve the antigens at 37 °C. Afterwards, 3% H2O2 was used to block endogenous peroxidase activity, and primary antibodies were used to incubate overnight under 4 °C. On the next day, each section was rinsed with Tris-buffered saline for 15 min, followed by further incubation using biotin-labeled goat anti-rabbit IgG (ZSGB-BIO, PV-6000). Later, diaminobenzidine (ZSGB-BIO Corp., China), a chromogenic substrate, was used for color development, and hematoxylin was used to counterstain the sections.

The Imaging Pro Plus 6.0 software (Media Cybernetics, Inc., USA) was used to calculate the ROI and to integrate the optical density (IOD). For measuring the mean IOD of certain proteins, we divided the total IOD by ROI, which was reported as IOD/mm^2^.

### Statistical analysis

SPSS20.0 (SPSS Inc.; Chicago, IL, USA) was applied in statistical analysis. Values were expressed in as mean ± SD. Normal distribution and homogeneity of variances were evaluated by Shapiro–Wilk and Bartlett’s test. One-way analysis of variance (ANOVA) and Fisher’s protected least significant difference (LSD) test were adopted for analyzing significant differences. Kruskal–Wallis test was applied in analyzing lumbar fusion scores. P < 0.05 suggested that a difference was of statistical significance.

## Results

### Manual palpation and X-ray analysis

**Fig. 1:**
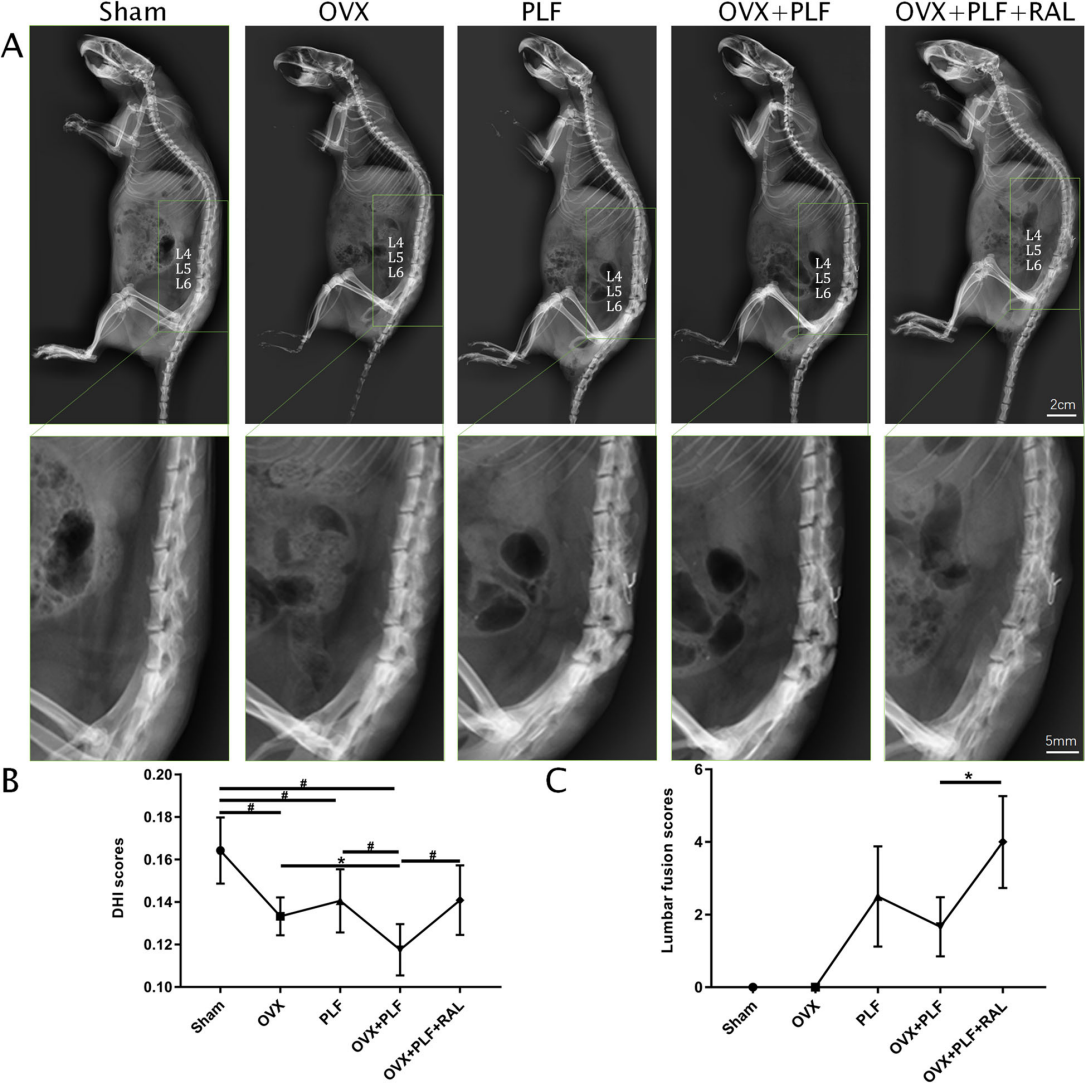
Radiographic images and analysis in all groups. **A** Typical radiographic images. **B** Intervertebral disk height (DHI) scores of L5–L6. **C** Lumbar fusion scores of L4–L5 segments. ^**#**^*p* < 0.01,^*^p < 0.05

Radiography was performed to observe the effects of DHI and lumbar fusion post-RAL treatment. According to Fig. 1, PLF, OVX, and OVX+PLF groups showed decreased DHI relative to sham group, whereas OVX+PLF+RAL group showed markedly increased DHI as compared with OVX+PLF groups. After manual palpation, there was no detectable movement at the fusion level. In contrast to the OVX+PLF group, those in the OVX+PLF+RAL group had elevated lumbar fusion scores. The difference was not significant in the PLF group relative to the OVX+PLF group.

### Histological examinations

**Fig. 2:**
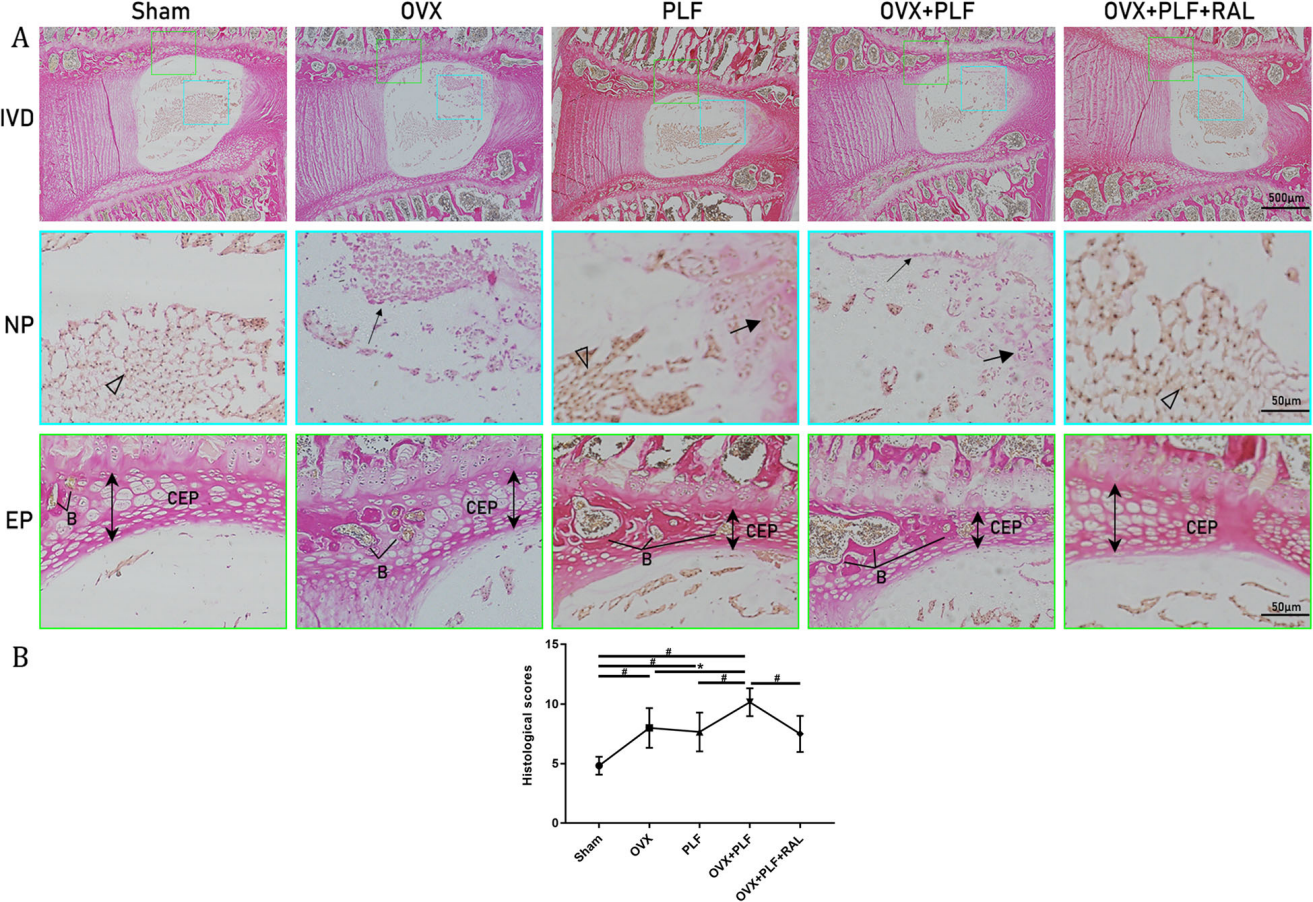
Van Gieson (VG) staining and histological scores of L5–L6 segments in each group. **A** Intervertebral disk degeneration (IVD), nucleus pulposus (NP) (notochord cells are indicated by the blank arrow, mucoid degeneration of NP is indicated by the thin arrow, and the doublets of chondrocyte-like cells are indicated by the large arrow), and endplate (EP). The double arrow represents the thickness of EP. CEP, cartilage endplate; VP, vertebral physis; B, bony tissues. **B** Histological scores. ^**#**^*p* < 0.01,^*^p < 0.05

To assess the function of RAL, we performed VG staining to observe adjacent segmental intervertebral disk and examine the histological structure. According to Fig. 2, compared with PLF, OVX, and OVX+PLF groups, certain notochord cells surrounded by rich ECM were observed in the sham group. AF was neatly arranged, and chondrocytes were observed within the EP. In addition, certain NP cells in PLF, OVX, and OVX+PLF groups were substituted by chondrocyte-like cell clusters. Mucoid degeneration to varying degrees was observed in the matrix surrounding the NP cells, with calcification within the EP. Aggravated degeneration was observed in the OVX+PLF group. But RAL treatment efficiently postponed such degenerative changes, as confirmed by histological score results.

### Micro-CT analysis of L6 vertebra

**Fig. 3:**
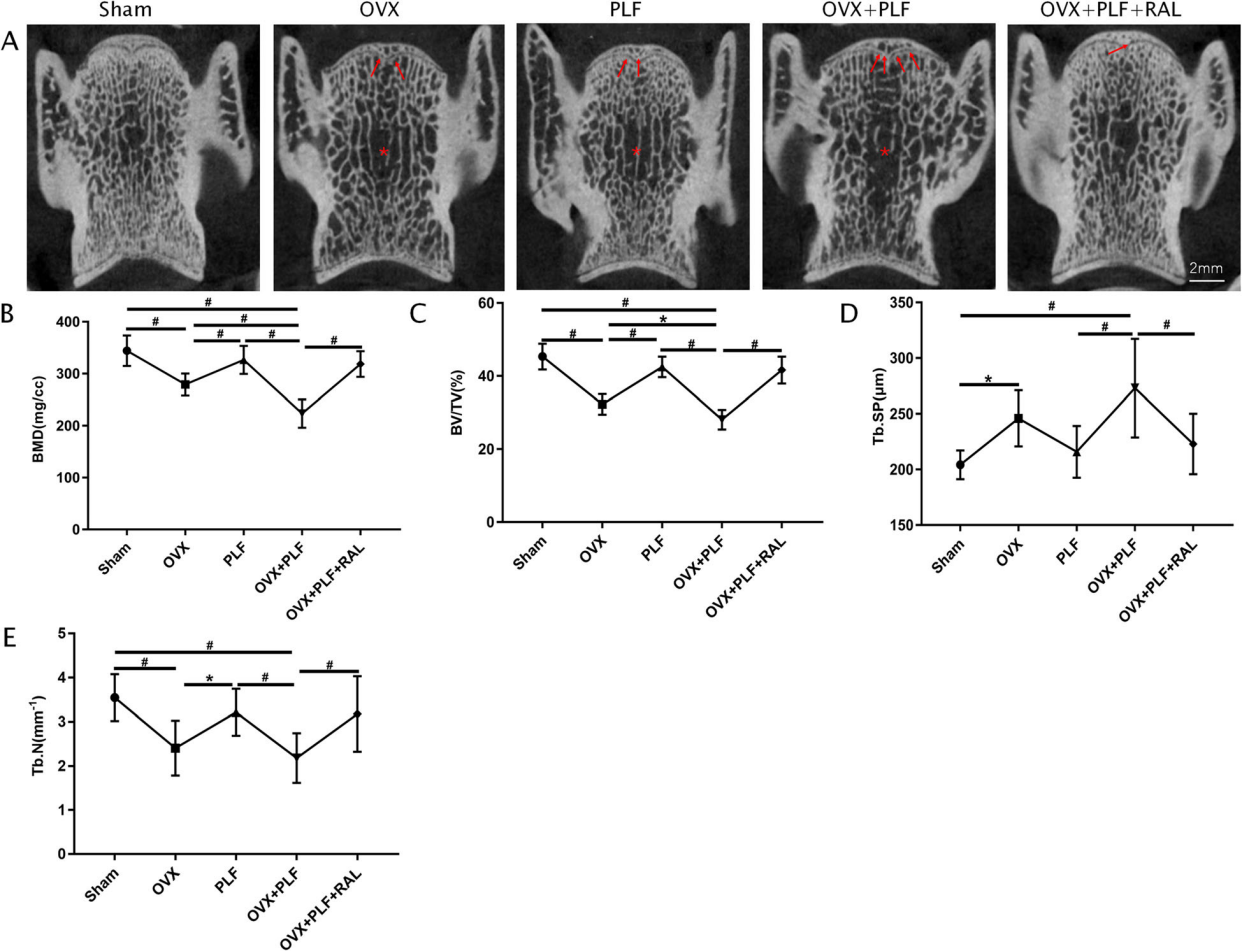
Micro-CT images and micro-architecture parameters of L6 were analyzed. **A**. Micro-CT images of L6 in all groups (the red arrow indicates trabecular cavities, the red asterisk indicates the sparse bone trabeculae). **B**–**E** Bone mineral density (BMD), bone volume fraction BV/TV, trabecular separation (Tb.Sp), and trabecular number (Tb.N). ^**#**^*p* < 0.01,^*^p < 0.05

According to Fig. 3, vertebral trabeculae and cavities were largely missing in PLF, OVX, and OVX+PLF groups. The RAL treatment markedly preserved the vertebral architecture. The micro-CT analysis confirmed that rats in the sham group exhibited significantly higher BV/TV, BMD, and Tb.N than those in other groups, but lower Tb.Sp relative to OVX and OVX+PLF groups. The OVX+PLF groups demonstrated higher BV/TV, BMD, and Tb.N but lower Tb.Sp relative to OVX+PLF+RAL groups. Nonetheless, the difference was not significant in the sham group compared with the PLF group. This confirmed that RAL delayed ASDD by maintaining the structure of the vertebra.

### Apoptosis examinations

**Fig. 4:**
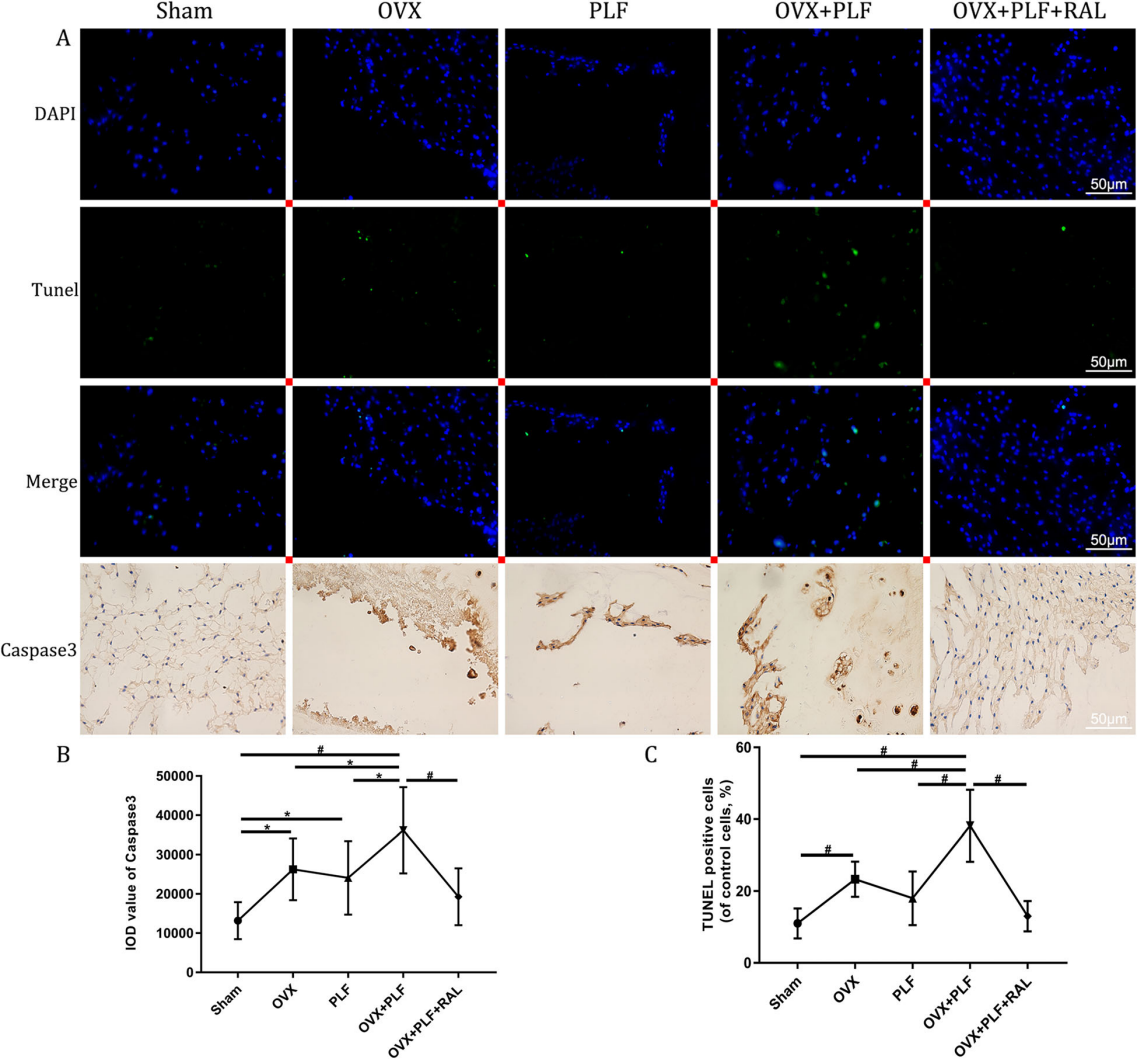
TUNEL staining and immunohistochemical assay for caspase 3 in each group. **A** TUNEL staining and staining of caspase 3 in the nucleus pulposus (NP). **B** IOD value of caspase 3 staining. **C** TUNEL positive cells. ^**#**^*p* < 0.01,^*^p < 0.05

**Fig. 5:**
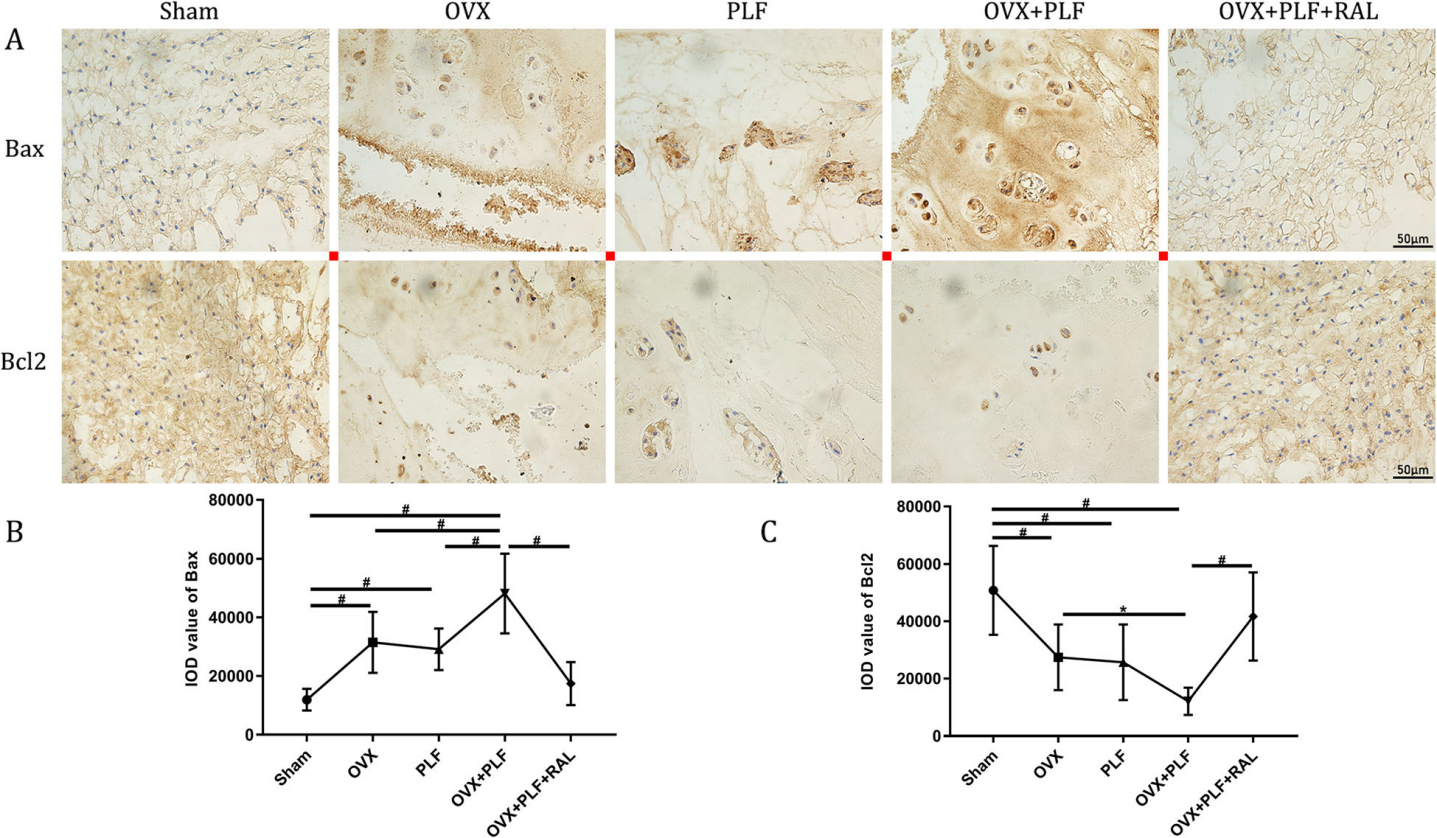
Immunohistochemistry assay of Bax and Bcl2 in the nucleus pulposus (NP) in all groups. ^**#**^*p* < 0.01, ^*^p < 0.05

To evaluate the apoptosis of NP, TUNEL and immunohistochemical staining were performed. Sham group showed markedly increased apoptosis and caspase 3 levels compared with those in the PLF, OVX, and OVX+PLF groups, whereas RAL exposure reduced apoptosis and declined caspase 3 and bax levels but increased bcl2 level relative to the OVX+PLF group (Figs. 4 and 5). These results verified the inhibitory effects of RAL on apoptosis in NP.

### Immunohistochemical assessments

**Fig. 6:**
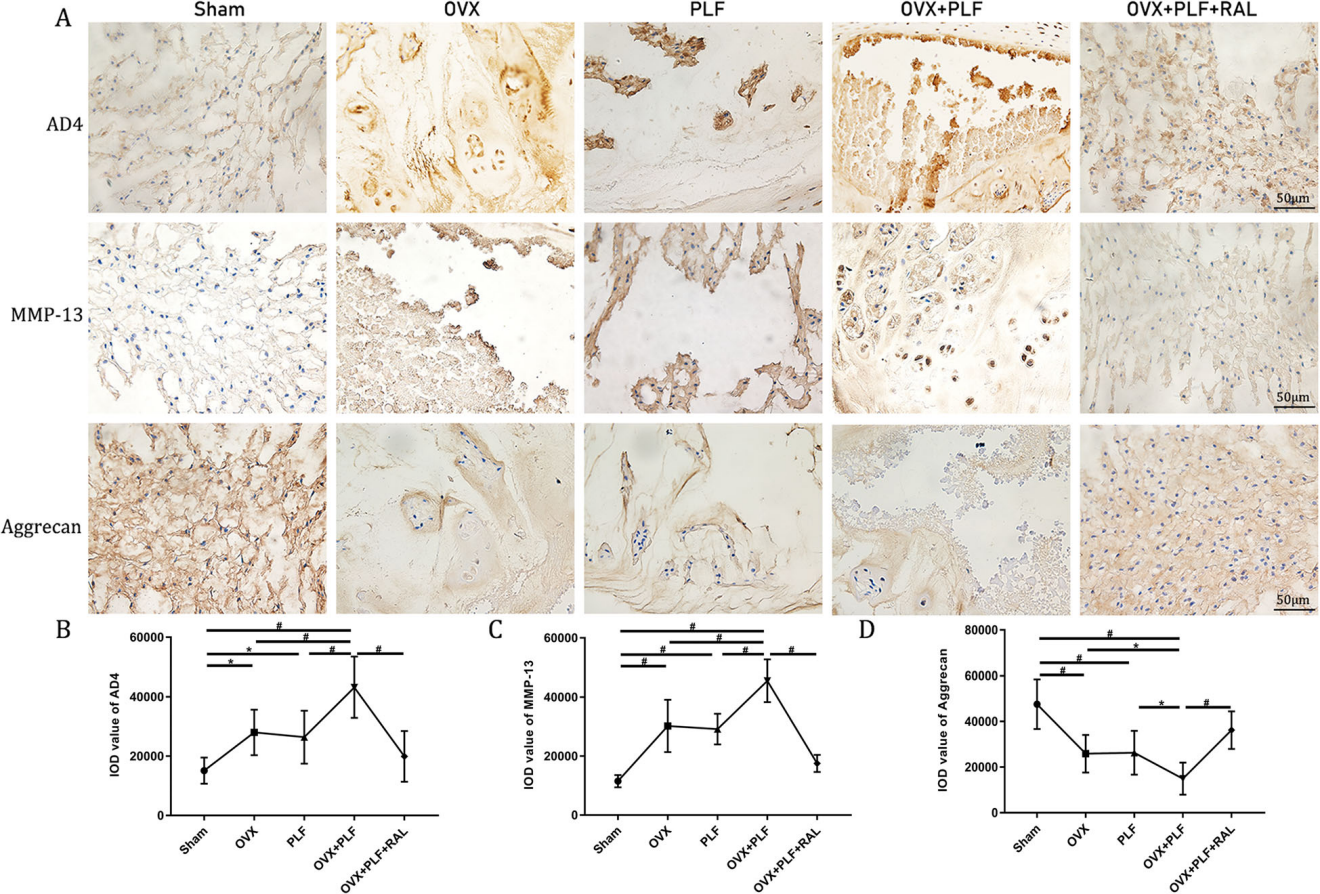
Immunohistochemistry assay of ADAMTS-4, MMP-13, and aggrecan in the nucleus pulposus (NP) in all groups. ^**#**^*p* < 0.01, ^*^p < 0.05

The aggrecan level was markedly reduced in PLF, OVX, and OVX+PLF groups as compared with that in the sham group. However, aggrecan level was elevated in the OVX+PLF+RAL group relative to the OVX+PLF group. MMP-13 and ADAMTS-4 levels markedly elevated in PLF, OVX, and OVX+PLF rats relative to the sham group, but declined in OVX+PLF+RAL groups compared with OVX+PLF group (Fig. 6). These findings verified that RAL protected against ECM in IVD.

## Discussion

Although the postoperative effect is unsatisfactory, surgery is the primary alternative for clinical treatment of ASDD. Because ASDD has high morbidity and limited current treatment modalities, studies on finding an effective therapeutic drug and exploring the pathogenesis of ASDD are urgently required. Our study showed that ASDD could be delayed by RAL in ovariectomized rats with lumbar fusion. Radiographic and micro-CT analyses revealed that RAL treatment preserved the vertebral microstructure and DHI. The above findings indicated that RAL might be used to treat ASDD.

Osteoporosis is characterized by reduced bone mass, bone microstructure destruction, and high fracture risk, which is more common in postmenopausal women. Some studies have confirmed that osteoporosis caused by estrogen deficiency can accelerate IVDD [20, 21]. For the simulation of this population, we performed OVX surgery to create a model of ASDD with osteoporosis in rats. According to Higashino et al. [22], BMD, DHI, and MRI signal intensity declined at 12 months post-spinal fusion in caudad. In this study, IVDD occurred in the adjacent disks after PLF, which was confirmed by a previous study [23]. In addition, the radiography and micro-CT analysis revealed that estrogen deficiency accelerated the L6 vertebral bone structural deterioration, postponed lumbar fusion, and decreased DHI of the L5–L6 segment. These destructive effects are inhibited by RAL treatment. In addition, the loss and degradation of ECM in the NP is an important pathological feature in IVDD. NP cells mainly synthesize ECM including collagen protein, proteoglycans, and water, which are the main components of the gelatinous tissues of NP [24]. They provide the ability for normal intervertebral disk resistance pressure. ADAMTS-4 is an aggrecan hydrolase, which accelerates the catabolism of aggrecan in the NP and further aggravates the degeneration of the intervertebral disk [25, 26]. MMP-13 can accelerate the degradation of the extracellular matrix, which plays an important role in IVDD [27]. In this study, we found that RAL could delay ASDD by downregulating the expression of ADAMTS4 and MMP13 but upregulating the expression of aggrecan.

Several studies have reported that estrogen deficiency exacerbates IVD, but estrogen supplementation hinders disk degeneration [28, 29]. Estrogen replacement therapy (ERT) can effectively inhibit the decrease in BMD caused by estrogen deficiency, improve the microstructure of bone trabecula, and reduce the fracture risks [21]. However, the application of ERT increases the risk of pituitary adenomas and reproductive cancers [30]. RAL, a SERM, not only supplements estrogen but also has no adverse effect of ERT [31]. Our results showed that relative to OVX+PLF group, OVX+PLF+RAL group showed elevated Tb.N, BMD, and BV/TV of the L6 segment and DHI. Following RAL treatment, increased osteolysis at the fusion site and increased radiographic density were observed in the L4–L5 segments group relative to the OVX+PLF group.

The generation of inflammatory factors, NP apoptosis, and ECM decomposition has been implicated in ASDD pathogenesis [32]. The apoptosis of NP may accelerate additional degeneration during IVDD [3]. Apoptosis leads to microenvironment transformation, disturbed nutrient absorption, and NP oxidative stress in the IVD, accelerating ASDD [33]. Chen et al. [34] verified the effects of metformin on reversing IVDD by suppressing NP apoptosis. Our results verified the markedly reduced ADAMT4, MMP-13, caspase 3 and bax levels, and TUNEL-positive cells but increased bcl 2 level among OVX+PLF group following RAL treatment, along with markedly elevated aggrecan levels in NP, suggesting that RAL suppressed NP apoptosis and ECM decomposition during ASDD. In this context, one limitation of the present study is that rat, as a reptile, is different from human being in terms of walking pattern. It is admitted that it cannot simulate the upright walking pattern of human beings, so the effects of human body weight on ASDD cannot be shown precisely. Therefore, a more suitable animal model is to be selected in future studies, thereby better simulating the physiological and pathological changes of human intervertebral disk.

## Conclusions

In summary, our study demonstrated that estrogen deficiency deteriorated ASDD condition by destroying the vertebral body structure. In addition, NP apoptosis and ECM degradation accelerated ASDD pathogenesis. RAL administration delayed ASDD progression, primarily by inhibiting vertebral osteoporosis, NP apoptosis, and ECM decomposition. These results can offer a new therapeutic option to treat ASDD.

## Data Availability

The datasets used and/or analyzed during the current study are available from the corresponding author on reasonable request.

## Abbreviations

RAL: Raloxifene
BMD: Bone mineral density
ASDD: Adjacent segmental intervertebral disk degeneration
Micro-CT: Microcomputed tomography
IVD: Intervertebral disk
IVDD: Intervertebral disk degeneration
DHI: Disk height index
OVX: Ovariectomy
PLF: Posterolateral lumbar fusion
Agg: Aggrecan
MMP-13: Metalloproteinase-13
ADAMTS-4: A disintegrin and metalloproteinase with thrombospondin motifs 4
bcl-2: B cell lymphoma-2
bax: BCL2-associated X
ROI: Region of interest
BV/TV: Bone volume fraction
Tb.N: Trabecular number
Tb.Sp: Trabecular separation
TUNEL: Transferase dUTP nick end labeling
AF: Annulus fibrosus
EP: Endplates
NP: Nucleus pulposus
VG: Van Gieson
ERT: Estrogen replacement therapy
ECM: Extracellular matrix
ROI: Region of interest
SERM: Selective estrogen receptor modulator
